# Cell Identity and Spatial Distribution of PD‐1/PD‐L1 Blockade Responders

**DOI:** 10.1002/advs.202400702

**Published:** 2024-09-09

**Authors:** Xintong Li, Yuanxin Liu, Jun Gui, Lu Gan, Jianxin Xue

**Affiliations:** ^1^ Division of Thoracic Tumor Multimodality Treatment State Key Laboratory of Biotherapy and Cancer Center National Clinical Research Center for Geriatrics West China Hospital Sichuan University Chengdu 610041 China; ^2^ State Key Laboratory of Systems Medicine for Cancer Renji‐Med X Clinical Stem Cell Research Center Ren Ji Hospital Shanghai Jiao Tong University School of Medicine Shanghai 200127 China; ^3^ Research Laboratory of Emergency Medicine Department of Emergency Medicine National Clinical Research Center for Geriatrics West China Hospital Sichuan University Chengdu 610041 China; ^4^ Division of Thoracic Tumor Multimodality Treatment State Key Laboratory of Biotherapy and Cancer Center National Clinical Research Center for Geriatrics Laboratory of Clinical Cell Therapy West China Hospital Sichuan University Chengdu 610041 China

**Keywords:** cancer, immunotherapy, PD‐1/PD‐L1 blockades, T‐cell exhaustion, stem‐like T‐cells, cell therapy

## Abstract

The programmed death 1 (PD‐1)/programmed death ligand 1 (PD‐L1) axis inhibits T cell activity, impairing anti‐tumor immunity. Blocking this axis with therapeutic antibodies is one of the most promising anti‐tumor immunotherapies. It has long been recognized that PD‐1/PD‐L1 blockade reinvigorates exhausted T (T_EX_) cells already present in the tumor microenvironment (TME). However, recent advancements in high‐throughput gene sequencing and bioinformatic tools have provided researchers with a more granular and dynamic insight into PD‐1/PD‐L1 blockade‐responding cells, extending beyond the TME and T_EX_ populations. This review provides an update on the cell identity, spatial distribution, and treatment‐induced spatiotemporal dynamics of PD‐1/PD‐L1 blockade responders. It also provides a synopsis of preliminary reports of potential PD‐1/PD‐L1 blockade responders other than T cells to depict a panoramic picture. Important questions to answer in further studies and the translational and clinical potential of the evolving understandings are also discussed.

## Graphical abstract

Systemic PD‐1/PD‐L1 blockade mainly works locally on tumor‐specific memory T cells in lymph nodes to promote their proliferation and differentiation into exhausted T cells in the TME, while many other cell types are also responsive to PD‐1/PD‐L1 blockade. These recent updates are promising to improve PD‐1/PD‐L1 blockade‐centered immunotherapies, cell therapy, and lymph node management strategies.

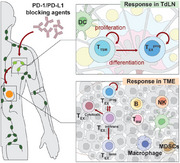

## 1 Introduction

Programmed death 1 (PD‐1) is an inhibitory receptor (IR) that is significantly induced upon T cell activation. When interacting with programmed death ligand 1 (PD‐L1), it suppresses multiple processes involved in T cell receptor (TCR) signaling.^[^ ^1^, ^2^ ^]^ PD‐L1 can also bind to B7‐1, competing with the costimulatory signal transmitted by CD28.^[^ ^3^ ^]^ The PD‐1/PD‐L1 axis plays an instrumental role in sustaining immune tolerance under physiological conditions^[^ ^4^, ^5^ ^]^ while mediating tumor immune evasion.

**Figure 1:**
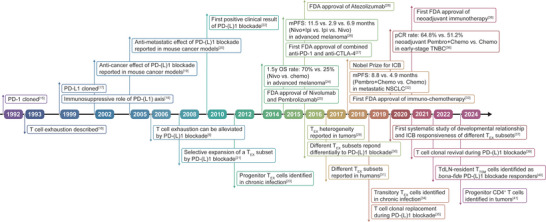
Timeline of Milestone Events in the Field of Programmed Death 1 (PD‐1)/Programmed Death Ligand 1 (PD‐L1) Blockade. Information about the preclinical and clinical development of PD‐1/PD‐L1 blockade is listed above the main axis, and information regarding the identification of the specific cell subpopulations responding to PD‐1/PD‐L1 blockade is listed below the main axis. Exhausted T (T_EX_), Food and Drug Administration (FDA), antibody (Ab), overall survival (OS), median progression‐free survival (mPFS), cytotoxic T lymphocyte antigen‐4 (CTLA‐4), Nivolumab (Nivo), Pembrolizumab (Pembro), Atezolizumab (Atezo), Ipilimumab (Ipi), chemotherapy (Chemo), immune checkpoint blockade (ICB), non‐small cell lung cancer (NSCLC), triple‐negative breast cancer (TNBC), pathological complete regression (pCR), tumor‐specific memory T (T_TSM_), tumor‐draining lymph node (TdLN).

Since the first discovery of PD‐1/PD‐L1 axis two decades ago as a negative regulator of T cell activity, PD‐1/PD‐L1 blocking antibodies and related combination therapies have achieved remarkable success in cancer patients^[^ ^6^, ^7^ ^]^ (**Figure** **1**). However, the specific cell population(s) that respond to anti‐PD‐1/PD‐L1 treatment remains unclear until recently (Figure 1). Serial high‐throughput profiling of the tumor tissues, peripheral blood, and lymph tissues at different time points has greatly advanced our understanding of PD‐1/PD‐L1 blockade responders. The long‐held view that pre‐existing intra‐tumoral exhausted T (T_EX_) cells are the primary cell subset responding to PD‐1/PD‐L1 blockade^[^ ^8^, ^9^ ^]^ has been greatly complemented. From the current perspective, PD‐1/PD‐L1 blockade can only provoke the response from stem‐like T cells, of which a substantial proportion are activated outside of the tumor microenvironment (TME). Furthermore, there is an increasing number of studies demonstrating that immunocytes other than the most‐studied T cells, and even tumor cells, can respond to PD‐1/PD‐L1 blockade.

Despite tremendous success, PD‐1/PD‐L1 blockade still faces several clinical challenges, including the lack of reliable predictors of patients’ response,^[^ ^10^ ^]^ treatment resistance even in immunologically “hot” tumors,^[^ ^11^, ^12^ ^]^ and the occurrence of systemic immune‐related adverse events.^[^ ^13^, ^14^ ^]^ Elucidating the identity of PD‐1/PD‐L1 blockade responders and understanding their response patterns is critical to a more accurate understanding of the mechanisms underlying treatment response and resistance, and holds promise in improving PD‐1/PD‐L1 blockade and other immuno‐oncology strategies.

In this review, we provide an update on the cellular response to PD‐1/PD‐L1 blockade, focusing on the specific cell (sub)populations that respond to PD‐1/PD‐L1 blockade, their molecular and functional characteristics, spatial distribution, and treatment‐induced spatiotemporal dynamics. Important research gaps to be filled in future studies and inspirations for improving clinical practice are also discussed.

## 2 PD‐1/PD‐L1 Blockade Responders in CD8^+^ T Cell Population

### 2.1 Overview of CD8^+^ T Cell Responses during Cancer

**Figure 2:**
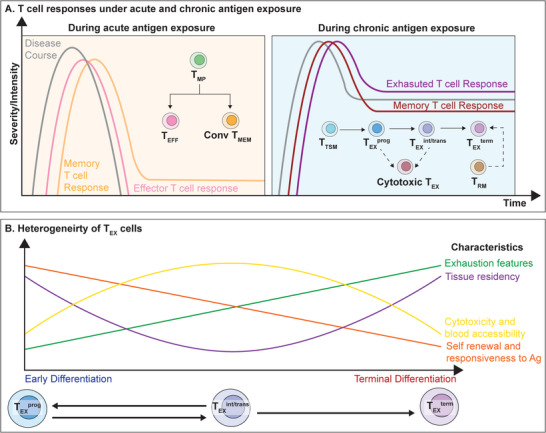
Acute and Chronic T cell Responses and Heterogeneity of Exhausted T (T_EX_) Cells. A) During acute antigen exposure (left panel), the disease is quickly controlled by effector T (T_EFF_) cells and memory T (T_MEM_) cells derived from memory precursor T (T_MP_) cells. Subsequently, T_EFF_ cells undergo apoptosis, while a low level of T_MEM_ cells persist. During chronic antigen exposure (right panel), the T cell response is mainly mediated by T_EX_ cells and both circulating tumor‐specific memory T (T_TSM_) cells and tissue‐resident memory T (T_RM_) cells and persists chronically. B) The T_EX_ subpopulations mainly consist of early differentiated progenitor exhausted T (T_EX_^prog^) cells, moderately differentiated intermediate/transitory exhausted T (T_EX_^int/trans^) cells, and terminally differentiated terminal exhausted T (T_EX_^term^) cells. The curve graph shows changes in phenotypic and functional characteristics along the differentiation process. Antigen (Ag).

During acute antigen exposure, such as acute infections and vaccinations (**Figure** **2**A, left panel), naïve CD8^+^ T cells clonally expand and differentiate into two distinct subsets of effector cells capable of cytotoxicity and cytokine production, known as KLRG1^hi^ CD127^lo^ terminal effector T (T_EFF_) cells and KLRG1^lo^ CD127^hi^ memory precursor T (T_MP_) cells.^[^ ^42^ ^]^ Following antigen clearance and resolution of inflammation, over 90% of T_EFF_ cells undergo apoptosis to avoid excessive immune response,^[^ ^43^ ^]^ while T_MP_ cells give rise to long‐lived memory T (T_MEM_) cells, establishing immune memory.^[^ ^42^ ^]^

Under circumstances with persistent antigen stimulation, such as chronic infections or tumors (Figure 2A, right panel), the main effector immune response is generated by a distinct subset of T_EX_ cells. T_EX_ cells represent a functionally impaired lineage that is transcriptionally and epigenetically distinct from T_EFF_ cells and T_MEM_ cells.^[^ ^44^, ^45^, ^46^ ^]^ They display crippled effector functions, progressive loss of proliferative capability, and stepwise upregulation of IR expression. T_EX_ cells acquire a fixed chromatin state during exhaustion known as “epigenetic scarring” early after T cell activation, which renders them hyporesponsive even after antigen elimination.^[^ ^44^, ^45^, ^46^ ^]^ The compromised yet critical immune response mounted by T_EX_ cells represents a delicate tipping point in balancing disease control and tissue damage during the persistent disease course.^[^ ^47^, ^48^, ^49^ ^]^ However, T‐cell exhaustion is also one of the major mechanisms employed by tumors to evade immune surveillance.

T_EX_ cells maintain a hierarchal organization, consisting of stem‐like progenitor exhausted (T_EX_^prog^) cells, intermediate/transitory exhausted T (T_EX_^int/trans^) cells, and terminal exhausted (T_EX_^term^) cells (Figure 2B). T_EX_^prog^ cells express higher levels of stem/memory‐related genes and exhibit greater persistence and recall response compared to T_EX_^term^ cells.^[^ ^21^, ^23^, ^30^, ^50^, ^51^, ^52^, ^53^ ^]^ They are also more polyfunctional regarding cytokine production, which corresponds to their lower degree of differentiation, while T_EX_^term^ cells show a superior ability to degranulate, produce granzymes and perforin, and perform in vitro cytolysis.^[^ ^21^, ^23^, ^30^, ^50^, ^52^, ^53^ ^]^ T_EX_^prog^ cells also contain heterogeneous subsets with different levels of proliferative and metabolic activity.^[^ ^34^, ^54^ ^]^ T_EX_^int/trans^ cells show an intermediate level of stem/memory‐like and exhaustion features and can transition between T_EX_^prog^ cells and T_EX_^term^ cells.^[^ ^37^, ^55^, ^56^ ^]^ Importantly, they display superior effector functions than both subsets.^[^ ^37^, ^55^, ^56^ ^]^ Markers used to delineate these T_EX_ subsets vary across studies (**Table** **1**). Generally, T_EX_^prog^ cells express higher levels of T‐bet,^[^ ^23^ ^]^ TCF1,^[^ ^50^, ^51^ ^]^ SLAMF6/Ly108 in mice (surface surrogates of TCF1), and CXCR5.^[^ ^29^, ^30^, ^40^, ^57^ ^]^ This hierarchal relationship was initially described in mouse models of lymphocytic choriomeningitis virus (LCMV) infection and later confirmed in mouse cancer models and patients with various cancer types.^[^ ^29^, ^57^, ^58^, ^59^, ^60^, ^61^, ^62^, ^63^, ^64^, ^65^ ^]^ Most studies propose a linear model in which T_EX_^prog^ cells self‐renew and differentiate into T_EX_^term^ cells through an intermediate/transitory state. Along the differentiation process, stem‐like features gradually diminish, and exhaustion features become more prominent. Notably, some studies suggest a branched trajectory in which either T_EX_^int/trans^ cells^[^ ^55^ ^]^ or T_EX_^prog^ cells^[^ ^54^, ^56^ ^]^ could differentiate into a more cytotoxic subset. This cytotoxic T_EX_ subset shares transcriptional, epigenetic, and proteomic features with T_EFF_ cells, and shows superior granzyme B production and direct killing capacity in vitro compared to the T_EX_ subsets described earlier.^[^ ^54^, ^55^, ^56^, ^66^ ^]^ Further studies are needed to explore the relationship between these two developmental paths.

**Table 1:** Exhausted T (T_EX_) subpopulations and markers from representative studies.

Time and Reference	Model/Patient	Nomenclature	Markers
2008^[^ ^21^ ^]^	LCMV Cl13 infection model	‐	PD‐1^lo^
		‐	PD‐1^int^ CD44^hi^
		‐	PD‐1^hi^ CD44^int^
2012^[^ ^23^ ^]^	LCMV Cl13 infection model	progenitor subset	T‐bet^hi^
		terminal subset	Eomes^hi^
2016^[^ ^51^ ^]^	LCMV Cl13 infection model	progenitor subset	TCF1^hi^ Tim3^lo^
		terminal subset	TCF1^lo^ Tim3^hi^
2016^[^ ^50^ ^]^	LCMV Cl13 infection model	progenitor subset	TCF1/Slamf6/Ly108^+^
		terminal subset	TCF1/Slamf6/Ly108^‐^
2016^[^ ^52^ ^]^	LCMV Cl13 infection model LCMV DOC infection model HIV‐infected patients	progenitor subset	CXCR5^+^
		terminal subset	CXCR5^‐^
2017^[^ ^58^ ^]^	tamoxifen‐induced liver cancer model B16 melanoma model	dysfunctional state 1	CD38^hi^ CD101^hi^
		dysfunctional state 2	CD38^lo^ CD101^lo^
2017^[^ ^31^ ^]^	chronic HCV‐infected patients		CD127^+^ PD‐1^‐^
			CD127^‐^ PD‐1^lo^
			CD127^‐^ PD‐1^hi^
2018^[^ ^59^ ^]^	NSCLC patients		PD‐1^T^
			PD‐1^N^
			PD‐1^‐^
2018^[^ ^64^ ^]^	melanoma patients	memory‐like	CD39^‐^ TIM3^‐^
		exhausted‐like	CD39^+^ TIM3^+^
2018^[^ ^60^ ^]^	NSCLC patients		CXCR5^+^ TIM3^‐^
			CXCR5^‐^ TIM3^‐^
			CXCR5^‐^ TIM3^+^
2019^[^ ^63^ ^]^	RCC patients	stem‐like CD8^+^ T cells	TCF1^+^ checkpoint^lo^
		terminally differentiated CD8^+^ T cells	TIM3^+^ checkpoint^hi^
2019^[^ ^46^ ^]^	LCMV Cl13 infection model	progenitor subset	KLRG1^‐^ PD^‐^1^+^ Ly108^+^
			KLRG1^‐^ PD^‐^1^+^ Ly108^‐^
2019^[^ ^80^ ^]^	LCMV Cl13 infection model B16 melanoma model	progenitor subset	Tcf1/Slamf6^+^ Tim3^‐^
		terminally exhausted subset	Tcf1/Slamf6^‐^ Tim3^+^
2019^[^ ^56^ ^]^	LCMV Cl13 infection model B16 melanoma model	progenitor CD8^+^ T cell	Ly108^+^ Marker TFs: TCF1, Id3
		exhausted CD8^+^ T cell	PD^‐^1^+^ CX3CR1^‐^ Ly108 Marker TFs: EOMES, Nr4a2
		cytolytic CD8^+^ T cell	CX3CR1^+^ Marker TFs: T^‐^bet, Zeb2
2019^[^ ^34^ ^]^	LCMV Cl13 infection model	stem‐like CD8^+^ T cells	PD^‐^1^+^ TCF1^+^
		transitory CD8^+^ T cells	CD101^‐^ Tim3^+^
		terminally differentiated CD8^+^ T cells	CD101^+^ Tim3^+^
2019^[^ ^81^ ^]^	B16 melanoma model		TCF1^+^ PD^‐^1^+^
			TCF1^‐^ PD^‐^1^+^
2020^[^ ^37^ ^]^	LCMV Cl13 infection model	T_ex_^prog1^	Ly108^+^ CD69^‐^
		T_ex_^prog2^	Ly108^+^ CD69^+^
		T_ex_^int^	Ly108^‐^ CD69^+^
		T_ex_^term^	Ly108^‐^ CD69^‐^
2020^[^ ^82^ ^]^	melanoma patients receiving TIL‐ACT	stem‐like (neoantigen‐specific) TIL	CD39^‐^ CD69^‐^
		terminally‐differentiated (neoantigen‐specific) TIL	CD39^+^ CD69^+^
2021^[^ ^39^ ^]^	NSCLC patients	proliferative T cells	
		progenitor exhausted T cells	GZMK^+^ NR4A2^‐^ cells
		progenitor exhausted T cells	GZMK^+^ NR4A2^+^ cells
		exhausted T cells	CXCL13^hi^
2022^[^ ^54^ ^]^	LCMV DOCILE infection model	T_pex_	PD^‐^1^+^ Tim3^‐^ CD62L^+^
		T_pex_	PD^‐^1^+^ Tim3^‐^ CD62L^‐^
		T_ex_	PD^‐^1^+^ Tim3^+^ CD62L^+^
		T_ex_	PD^‐^1^+^ Tim3^+^ CD62L^‐^
2022^[^ ^55^ ^]^	LCMV Cl13 infection model	T_ex_^prog^	PD^‐^1^+^ CX3CR1^‐^ SLAMF6^+^
		T_ex_^int^	PD^‐^1^+^ CX3CR1^+^ SLAMF6^‐^ KLRG1^‐^
		T_ex_^KLR^	PD^‐^1^+^ CX3CR1^+^ SLAMF6^‐^ KLRG1^+^
		T_ex_^term^	PD^‐^1^+^ CX3CR1^‐^ SLAMF6^‐^
2022^[^ ^40^ ^]^	B16 melanoma model	TdLN‐T_TSM_	TCF1^+^ TOX^‐^
		TdLN‐T_PEX_ and TME‐T_PEX_	TCF1^+^ TOX^+^
		TME‐T_EX_	TCF1^‐^ TOX^+^
2023^[^ ^83^ ^]^	B16‐OVA melanoma model Melanoma patients LCMV Cl13 infection model Healthy donors (EBV‐specific T cells)	T_pex_ T_ex_	TCF1^+^ TIM^‐^3^‐^ PD^‐^1^+^ CD8^+^ TCF1^‐^ TIM^‐^3^+^ PD^‐^1^+^ CD8^+^
2023^[^ ^84^ ^]^	B16‐OVA melanoma model	T_pex_1 T_pex_2 T_ex_1 T_ex_2	Tox^+^ Tcf7^+^ Havcr2^‐^ Ki67^‐^ Tox^+^ Tcf7^+^ Havcr2^‐^ Ki67^+^ Tox^+^ Tcf7^‐^ Havcr2^+^ Ki67^+^ Tox^+^ Tcf7^‐^ Havcr2^+^ Ki67^‐^

The table summarizes the animal models/patient populations, subsets of T_EX_ cells, and markers delineating these subsets in selected studies regarding the heterogeneity of T_EX_ cells. Lymphocytic choriomeningitis virus clone 13 (LCMV Cl13), lymphocytic choriomeningitis virus DOCILE clone (LCMV DOC), human immunodeficiency virus (HIV), hepatitis C virus (HCV), non‐small cell lung cancer (NSCLC), renal cell carcinoma (RCC), transcription factor (TF), tumor‐infiltrating lymphocyte (TIL), adoptive cell transfer (ACT), Epstein‐Barr virus (EBV).

In the context of immune memory, it was conventionally believed that chronic infection and cancer preclude the formation of T_MEM_ cells^[^ ^67^, ^68^ ^]^ until the recent discovery of tumor‐specific memory T (T_TSM_) cells in tumor‐draining lymph nodes (TdLNs) (Figure 2A, right panel). T_TSM_ cells have not committed to the exhaustion lineage but could differentiate into and sustain the T_EX_ population in TdLNs and the TME.^[^ ^40^, ^69^ ^]^ They show robust antigen‐independent self‐renewal and antigen‐dependent recall capabilities comparable to T_MEM_ cells generated during acute viral infection and superior to T_EX_ cells.^[^ ^40^, ^69^ ^]^ Additionally, a subset of T_EX_ cells has been observed to possess significant stem/memory‐like characteristics.^[^ ^67^ ^]^ Their ability to proliferate and produce inflammatory cytokines is only slightly weaker than stem cell‐like memory T (T_SCM_) cells and central memory T (T_CM_) cells.^[^ ^67^ ^]^ T_EX_ cells also express markers of conventional circulating memory T cells (T_SCM_: CCR7^+^ CD45RO^‐^ CD95^+^, T_CM_: CCR7^+^ CD45RO^+^ CD95^+^).^[^ ^67^ ^]^ Thus, even under chronic antigen exposure, certain T cells, whether exhausted or not, show memory features and may be considered memory cells.

In addition to circulating memory cells that recirculate between lymph organs and peripheral tissues, tissue‐resident memory T (T_RM_) cells that primarily patrol peripheral sites also contribute to anti‐tumor immune memory (Figure 2A, right panel). T_RM_ cells express tissue retention markers CD49a, CD69, and CD103 while lacking the expression of lymph‐homing receptor CD62L and the transcription factor (TF) TCF1,^[^ ^70^ ^]^ distinguishing them from the aforementioned T_TSM_ and T_EX_^prog^ cells. T_RM_ cells contribute substantially to the intra‐tumoral CD8^+^ T cell population (CD103^+^, ≈70%^[^ ^57^, ^71^, ^72^ ^]^; CD69^+^ CD103^+^, ≈30%^[^ ^73^, ^74^, ^75^ ^]^), and are enriched with tumor reactivity.^[^ ^55^, ^71^, ^76^, ^77^ ^]^ Notably, intra‐tumoral T_RM_ cells are not spared from exhaustion. They express intermediate levels of exhaustion‐associated markers and TFs^[^ ^62^, ^71^, ^77^, ^78^ ^]^ and are functionally impaired compared to T_RM_ cells in healthy tissues.^[^ ^79^ ^]^ The relationship between T_RM_ cells and T_EX_ cells, including whether T_RM_ cells display an exhausted scenario similar to circulating memory T cells and whether they also contribute to the reported T_EX_ cells, requires further investigation.^[^ ^61^, ^65^ ^]^

### 2.2 Stem‐like TEX^prog^ Cells as PD‐1/PD‐L1 Blockade Responders

**Figure 3:**
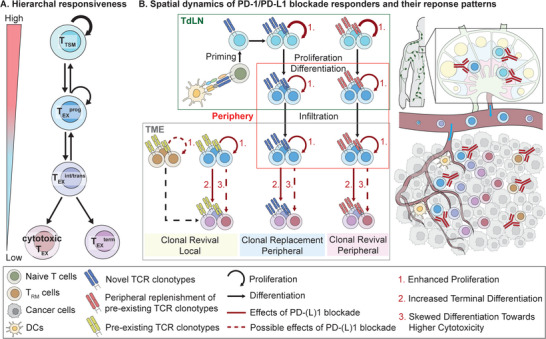
Main Programmed Death 1 (PD‐1)/Programmed Death Ligand 1 (PD‐L1) Blockade Responders and Their Spatial Distribution and Dynamics During Treatment. A) It illustrates that major subsets of tumor‐specific T cells show a progressive decline in responsiveness to PD‐1/PD‐L1 blockade along differentiation. B) The left panel shows that PD‐1/PD‐L1 blockade mainly enhances the proliferation of stem‐like T cells in tumor‐draining lymph nodes (TdLNs), which subsequently differentiate into more exhausted cells and infiltrate the tumor microenvironment (TME). Three main effects of PD‐1/PD‐L1 blockade on responding cells are indicated by red arrows: 1) Promotion of proliferation, 2) Facilitation of differentiation, and 3) Modulation of differentiation path. The expansion of intra‐tumoral T cells could be categorized based on their spatial origin (peripheral replenishment vs. local expansion) and the novelty of T cell receptor (TCR) clonotypes (clonal replacement vs. clonal revival). The right panel provides a more visual illustration of the spatial dynamics of immune cells during PD‐1/PD‐L1 blockade. Tumor‐specific T (T_TSM_), progenitor exhausted T (T_EX_^prog^), intermediate/transitory exhausted T (T_EX_^int/trans^), terminally exhausted T (T_EX_^term^), tissue‐resident memory T (T_RM_), dendritic cells (DCs).

Among T_EX_ subpopulations, PD‐1/PD‐L1 blockade preferentially expands T_EX_^prog^ cells, which corresponds to their higher proliferative ability (**Figure** **3**A). Transfer experiments have shown that only T_EX_^prog^ cells can expand during PD‐1/PD‐L1 blockade, and only the transfusion of T_EX_^prog^ cells could enhance disease control.^[^ ^21^, ^30^, ^52^, ^54^ ^]^ Beltra JC, et al. demonstrated that although the intermediate subset shows the most significant expansion during PD‐1 blockade, truly proliferating responders are the progenitor cells.^[^ ^37^ ^]^ Consistently, CD62L^+^ T_EX_^prog^ cells, which represent a quiescent T_EX_^prog^ subpopulation with high proliferative potential, exhibit the most significant proliferation in response to PD‐1/PD‐L1 blockade, exceeding CD62L^‐^ T_EX_^prog^ cells (an actively proliferating subpopulation) and T_EX_^term^ cells.^[^ ^54^ ^]^ These observations suggest that less differentiated cells expand more greatly during PD‐1/PD‐L1 blockade compared to their more differentiated counterparts, leading to an increase in the latter.

In addition to promoting the proliferation of stem‐like T_EX_ cells (Figure 3B, left panel), PD‐1/PD‐L1 blockade promotes their terminal exhaustion and may generate a more cytotoxic subset (Figure 3B, left panel). PD‐1 blockade has been shown to increase the conversion of virus‐specific CXCR5^+^ T_EX_^prog^ cells to CXCR5^‐^ T_EX_^term^ cells (CXCR5^‐^/CXCR5^+^ ratio: 3.2 vs. 35.2) in a transfer experiment in chronically infected mice.^[^ ^30^ ^]^ The increased terminal conversion has also been observed in tumors, where PD‐1 blockade increases the proportion of TCF1^+^ Tim3^‐^ T_EX_^prog^ cells converting to the TCF1^‐^ Tim3^+^ T_EX_^term^ counterpart (57% vs. 67%).^[^ ^80^ ^]^ Furthermore, PD‐1/PD‐L1 blockade may affect the differentiation path of T_EX_ cells (Figure 3B, left panel). Anti‐PD‐1 treatment may skew the differentiation of T_EX_^prog^ cells toward a more cytotoxic terminal state, generating a subset of CXCL13^+^ “better effectors”.^[^ ^85^ ^]^ This phenomenon has only been observed in responsive patients, indicating its importance for the efficacy of PD‐1/PD‐L1 blockade.^[^ ^85^ ^]^ However, it is reported that only when combined with cis IL‐2R agonism can PD‐1 blockade enhance the effector function of TCF1^+^ T_EX_^prog^ cells.^[^ ^86^ ^]^ Although this discrepancy may be attributed to different tumor models and experimental settings, it implies the need for further confirmation of the effects of PD‐1/PD‐L1 blockade and influencing factors.

The specific T_EX_ subset that shows the most significant accumulation in response to PD‐1/PD‐L1 blockade can vary across studies and cancer types. Liu B, et al. compared the pre‐ and post‐treatment CD8^+^ T cells based on single‐cell RNA sequencing (scRNA‐seq) datasets from different tumor types and demonstrated that PD‐1/PD‐L1 blockade induces increased CXCL13^+^ T_EX_^prog^ cells in responsive melanoma and non‐small cell lung cancer (NSCLC), but not in responsive skin basal cell carcinoma (BCC) and squamous cell carcinoma (SCC), where T_EX_^term^ cells accounted for the majority of post‐treatment tumor‐infiltrating T cells.^[^ ^39^ ^]^ This variable pattern may be attributed to different levels of immunosuppression in the TME. When immunosuppression is low (as assessed by the expression scores of IRs), PD‐1/PD‐L1 blockade is more likely to induce an increase in progenitor cells, whereas it induces more terminally differentiated cells when immunosuppression is high.^[^ ^39^ ^]^ Consistent with this, in chronic infections, which are less immunosuppressive and have a less exhausted pool of antigen‐specific T cells compared to tumors, T_EX_^prog^ cells are the subset that displays the most significant increase in proportion and number during PD‐1/PD‐L1 blockade.^[^ ^34^, ^37^ ^]^ Similar results are observed in tumors with low‐level immunosuppression, such as melanoma and NSCLC.^[^ ^39^ ^]^ Furthermore, different treatments may contribute to these variable patterns. It was observed in three breast cancer cohorts that patients receiving chemotherapy and anti‐PD‐1 treatment showed an increased proportion of T_EX_^prog^ cells, while patients receiving single‐agent anti‐PD‐1 treatment showed scarce and unchanged T_EX_^prog^ cells in post‐treatment responsive tumors.^[^ ^87^ ^]^ This could be explained by the augmented T cell priming and generation of T_EX_^prog^ cells induced by enhanced tumor immunogenicity through increased antigen expression^[^ ^88^ ^]^ and release^[^ ^89^ ^]^ as well as enhanced major histocompatibility complex (MHC)‐I‐mediated antigen presentation.^[^ ^90^ ^]^

Although T_EX_^prog^ cells are crucial for sustaining anti‐tumor immunity and mediating response toward PD‐1/PD‐L1 blockade, defective terminal differentiation of stem‐like CD8^+^ T cells may limit endogenous anti‐tumor immunity^[^ ^91^ ^]^ and the efficacy of tumor vaccine.^[^ ^92^ ^]^ Thus, it is essential to find the optimal balance between sustainability and magnitude of anti‐tumor response mediated by less‐ and more‐differentiated T_EX_ cells, respectively. Furthermore, exploring the differential effects of other therapeutic agents and endogenous factors on different subsets is an important area of investigation.

### 2.3 T_MEM_ Cells as PD‐1/PD‐L1 Blockade Responders

Recent studies have emphasized the importance of circulating and tissue‐resident memory responses in effective anti‐tumor immunity,^[^ ^93^ ^]^ both of which could be enhanced by immune checkpoint blockade (ICB)^[^ ^94^, ^95^ ^]^ (Figure 3B, left panel).

Circulating memory T cells possess the greatest potential in responding to PD‐1/PD‐L1 blockade. Huang Q, et al. reported that cells that expand most significantly during treatment are the non‐exhausted T_TSM_ cells, which show greater responsiveness than T_EX_^prog^ cells, the focus of previous studies.^[^ ^40^, ^69^ ^]^ When an equal number of TCF1^+^ TOX^‐^ T_TSM_ cells and TCF1^+^ TOX^+^ T_EX_^prog^ cells were transferred into tumor‐bearing mice receiving anti‐PD‐L1 treatment, TCF1^+^ TOX^‐^ T_TSM_ cells showed the most pronounced expansion.^[^ ^40^ ^]^ Moreover, within the second tumor transplanted into mice that had received PD‐L1 blockade and adoptive transfer of either T_TSM_ cells or T_EX_^prog^ cells, T_TSM_ cells exhibited a more significant expansion than T_EX_^prog^ cells when compared to pre‐existing tumor‐infiltrating T cells.^[^ ^40^ ^]^ On the other hand, some T_EX_^prog^ cells, as mentioned earlier, are highly similar to T_MEM_ cells.^[^ ^67^ ^]^ However, further exploration is needed to investigate which subset of T_EX_ cells can be considered bona fide memory cells to accurize our understanding of the memory response toward cancer.

T_RM_ cells, which express PD‐1 and become dysfunctional in the TME,^[^ ^62^, ^71^, ^77^, ^78^, ^79^ ^]^ may also be reinvigorated by PD‐1/PD‐L1 blockade. Accumulation of CD103^+^ CD8^+^ T cells has been observed following PD‐1/PD‐L1 blockade in melanoma,^[^ ^74^ ^]^ NSCLC,^[^ ^71^, ^78^ ^]^ and metastatic vaginal melanoma patients^[^ ^96^ ^]^ as well as in murine liver cancer,^[^ ^97^ ^]^ esophageal cancer,^[^ ^98^ ^]^ and breast cancer models.^[^ ^73^ ^]^ CD8^+^ T cells responsive to neoadjuvant PD‐1/PD‐L1 blockade have been reported to express a tissue‐resident memory gene program,^[^ ^78^, ^95^ ^]^ which is further enriched during treatment.^[^ ^95^ ^]^ The predictive value of the frequency or density of T_RM_ cells and T_RM_ signature also suggests the responsiveness of T_RM_ cells.^[^ ^71^, ^73^, ^74^, ^99^, ^100^ ^]^ Notably, increased T_RM_ cells may have two possible origins: 1) tissue‐resident cells that are already present in the local microenvironment before tumor development, and 2) circulating cells that infiltrate the TME and subsequently differentiate into T_RM_ cells.^[^ ^94^, ^101^ ^]^ Similar to circulating memory T cells, T_RM_ cells are heterogeneous, displaying different levels of immune checkpoint expression and differentiation.^[^ ^72^, ^78^, ^102^, ^103^ ^]^ In ovarian cancer patients, T_RM_‐like tumor‐infiltrating lymphocytes (TILs) follow a differentiation trajectory from stem‐like to terminally exhausted cells.^[^ ^76^ ^]^ Consistently, Blimp1^lo^ Id3^hi^ CD8^+^ T cells and Blimp1^hi^ Id3^lo^ CD8^+^ T cells with tissue‐resident gene signatures respectively share transcriptomic similarities with memory‐like cells (e.g., T_CM_, T_MP_, and T_EX_^prog^ cells) and terminally differentiated effector‐like cells (e.g., T_EM_, T_EFF_, and T_EX_^term^ cells).^[^ ^104^ ^]^ Therefore, it is likely that T_RM_ subsets with relatively more stem/memory‐like characteristics may have a higher responsiveness to PD‐1/PD‐L1 blockade. However, current understanding has been limited regarding the exact origin of intra‐tumoral T_RM_ cells, their heterogeneity, and how they may respond to ICB, suggesting the need for further investigation.

## 3 Spatial Distribution and Spatial Dynamics of Responding T Cells

The site of action of PD‐1/PD‐L1 blockade is a crucial aspect of the cellular response and has been greatly updated in recent years. Systemic immune activation originated from TdLNs during PD‐1/PD‐L1 blockade has gained increasing recognition (Figure 3B), supported by several lines of evidence: 1) preferential lymph node (LN) residency of responding T cells, 2) importance of LNs in treatment efficacy, 3) proliferating T cells captured in peripheral blood post‐treatment, and 4) emergence of novel TCR clonotypes in the TME during treatment.

### 3.1 Preferential Lymph Residency of Responding T Cells

T_EX_^term^ cells, which constitute the largest proportion of intra‐tumoral tumor‐reactive T cells, can hardly be reinvigorated.^[^ ^45^, ^105^, ^106^, ^107^, ^108^ ^]^ Meanwhile, T_TSM_ cells and T_EX_^prog^ cells, major responders to PD‐1/PD‐L1 blockade, are strictly^[^ ^40^, ^69^ ^]^ and preferentially resident in LNs,^[^ ^56^, ^59^, ^61^ ^]^ respectively.

Notably, certain LN‐related cellular structures within the TME are associated with the localization of T_EX_^prog^ cells, supporting the notion of preferential lymph residency of stem‐like T cells. T_EX_^prog^ cells are reported to co‐localize with antigen‐presenting cells (APCs) in the TME.^[^ ^63^, ^81^, ^109^ ^]^ Such APC niches resemble the T‐cell zone in LNs, where naïve T cells are primed by dendritic cells (DCs).^[^ ^66^ ^]^ Many studies have further characterized APCs in such cellular structures. MregDCs, which express high levels of maturation‐related, costimulatory, and MHC‐II molecules, have been found to enrich near CD8^+^ T_EX_^prog^ cells in human liver cancer^[^ ^85^ ^]^ and lung cancer.^[^ ^110^ ^]^ They enable the optimal priming of CD8^+^ T cells and express high levels of cytokines that promote the survival of CD8^+^ T cells.^[^ ^85^ ^]^ Another study reported the presence of type 1 conventional dendritic cells (cDC1s) near TCF1^+^ stem‐like CD8^+^ T cells.^[^ ^111^ ^]^ These cDC1s also express high levels of MHC‐II molecules and secrete T cell‐chemo‐attractants CXCL9 and CXCL10, promoting T‐cell response.^[^ ^111^ ^]^ In addition to APCs, the optimal priming of CD8^+^ T cells within LNs requires help from CD4^+^ T cells.^[^ ^112^ ^]^ Correspondingly, CD4^+^ T cells have been reported as critical components of stem‐like T cell‐related structures. In hepatocellular carcinoma patients receiving anti‐PD‐1 treatment, CXCL13^+^ helper T (T_H_) cells are arranged in close proximity with CD8^+^ T_EX_^prog^ cells and mregDCs, especially in responsive patients.^[^ ^85^ ^]^ They can sustain the survival of CD8^+^ T_EX_ cells and produce chemo‐attractants for T_EX_^prog^ cells.^[^ ^85^ ^]^ A recent study suggested that intra‐tumoral immune cell triads involving CD4^+^ T cells, CD8^+^ T cells, and DCs are indispensable for effective ICB and T cell transfer therapy.^[^ ^113^ ^]^ CD4^+^ T cells within the triad reprogram CD8^+^ T cells toward a progenitor‐like state, alleviating exhaustion and enhancing cytotoxicity of intra‐tumoral CD8^+^ T cells.^[^ ^113^ ^]^ Nevertheless, it is also reported that the proliferation and phenotype of T_EX_^prog^ cells remain unaffected in the absence of CD4^+^ T cell help.^[^ ^114^ ^]^ These divergent findings suggest the potential heterogeneity of intra‐tumoral T‐cell zone‐like structures. CCL19^+^ fibroblasts are recognized as markers of some APC niches with putative interactions with CD4^+^ T cells and B cells.^[^ ^110^ ^]^ Similarly, fibroblastic reticular cells are critical to the organization and function of the T‐cell zone.^[^ ^115^ ^]^

Tertiary lymphoid structures (TLS) are B‐cell follicle‐like immune cell aggregates found in tumors.^[^ ^116^ ^]^ Intra‐tumoral CD8^+^ T cells are frequently located inside or surrounding TLS,^[^ ^59^, ^117^, ^118^ ^]^ especially the stem‐like TCF1^+^ CD8^+^ T cells.^[^ ^81^ ^]^ Mechanistically, tumor‐reactive CD8^+^ T cells express TLS‐forming CXCL13,^[^ ^87^ ^]^ and T_EX_^prog^ cells are characterized by the expression of the CXCL13 receptor, CXCR5.^[^ ^116^ ^]^ As tumor‐reactive CD8^+^ T cells become exhausted, they upregulate CXCL13 expression and initiate TLS formation. Reciprocally, TLS recruits and maintains CXCR5^+^ T_EX_^prog^ cells, forming a positive feedforward loop.

Overall, studies have provided preliminary insights into the cellular crosstalk within these cellular structures responsible for the presence of T_EX_^prog^ cells. Factors facilitating the formation of these structures and how cellular interactions promote the generation and maintenance of stem‐like T cells are areas worthy of investigation.

### 3.2 Importance of LNs to PD‐1/PD‐L1 Blockade‐invoked Anti‐Tumor Immunity

Surgical removal of TdLNs or pharmacologically blocking T cell egression from TdLNs abolishes the efficacy of PD‐1/PD‐L1 blockade, even in mouse tumor models that are sensitive to immunotherapy.^[^ ^119^, ^120^, ^121^ ^]^ Interestingly, the diminished immune activation and therapeutic efficacy can be reproduced by the adoptive transfer of lymph‐resident T_TSM_ cells.^[^ ^119^ ^]^ Selective administration of anti‐PD‐1/PD‐L1 agents to TdLNs induces immune reprogramming in the TME and tumor control comparable to systemic administration.^[^ ^122^ ^]^ In three different mouse models of melanoma and breast cancer, intradermal administration targeting TdLNs results in higher intra‐tumoral concentrations of anti‐PD‐1 antibodies, elevated T cell responses in both primary and secondary tumors, and enhanced tumor growth control than systemic administration.^[^ ^123^ ^]^ Similarly, both intraperitoneal and intranodal injection of anti‐PD‐L1 antibodies effectively suppress tumor growth, whereas intra‐tumoral injection has shown limited efficacy.^[^ ^40^ ^]^ The importance of LNs in generating a systemic anti‐tumor immune response has also been proposed in other anti‐tumor therapeutic modalities, including chemotherapy‐induced immunogenic cell death and antigen spread^[^ ^124^ ^]^ and radiotherapy‐induced abscopal effects.^[^ ^125^, ^126^ ^]^

Specifically, DCs in TdLNs emerge as critical mediators of LN‐dependent immune activation.^[^ ^127^, ^128^ ^]^ As demonstrated by longitudinal sampling and scRNA‐seq, cDC1s maintain a reservoir of TCF1^+^ CD8^+^ T cells within the LNs, and the frequency of cDC1s correlates with the strength of anti‐tumor immune response.^[^ ^121^ ^]^ Although both macrophages and DCs express high levels of PD‐L1 in LNs, only the depletion of DCs or selective deletion of PD‐L1 in DCs significantly impairs the efficacy of anti‐PD‐L1 treatment.^[^ ^122^, ^129^ ^]^

These findings further underscore LNs as important immune base stations in mounting effective anti‐tumor immune responses and response to PD‐1/PD‐L1 blockade.

### 3.3 T Cell Proliferative Response in Peripheral Blood

The immune activation detected in peripheral blood can predict treatment response in patients receiving ICB. The peripheral T cell proliferative response early after treatment, as reflected by the proportion of Ki67^+^ CD8^+^ T cells,^[^ ^130^ ^]^ TCR diversity and T cell clone size,^[^ ^131^ ^]^ and the number of neoantigen‐reactive CD8^+^ T cells,^[^ ^132^ ^]^ correlates with patients’ response to PD‐1 blockade. Gene signatures of clones that exhibit parallel expansion in both the blood and TME are associated with an improved response to anti‐PD‐1 treatment.^[^ ^102^ ^]^

Neoantigen‐specific CD8^+^ T cells are enriched in the peripheral circulation during ICB,^[^ ^133^, ^134^ ^]^ and T cell proliferation dynamics in the peripheral blood have been documented. In a patient who experienced a strong cellular and pathological response to neoadjuvant immunotherapy, the abundance of treatment‐responsive T cells peaked two weeks after treatment initiation in circulation and gradually decreased to an undetectable level, possibly due to T cell homing to malignant tissues where they exert their cytotoxic functions.^[^ ^95^ ^]^ A similar dynamic has been observed in an NSCLC patient who experienced a pathological complete response after neoadjuvant anti‐PD‐1 treatment, with a transient peripheral T‐cell expansion.^[^ ^78^ ^]^ The proportion of common TCR clonotypes between the TME and peripheral blood also significantly increases two to four weeks after the initiation of neoadjuvant anti‐PD‐1 treatment, and the proportion of shared clonotypes correlates with the patient's pathological response.^[^ ^135^ ^]^

These observations suggest that the peripheral blood may serve as an important route for the anti‐tumor immune response induced by PD‐1/PD‐L1 blockade to reach the TME.

### 3.4 Novel T Cells and TCR Clonotypes Replenished from Blood into the TME during PD‐1/PD‐L1 Blockade

The origin of intra‐tumoral treatment‐expanded T cells can be attributed to two possible sources: 1) local expansion of pre‐existing T cells and 2) replenishment with peripheral T cells (Figure 3B, left panel).

After labeling peripheral T cells with 5‐Bromo‐2‐deoxyUridine (BrdU), accumulation of BrdU^+^ T cells within the TME has been observed after anti‐PD‐L1 treatment,^[^ ^40^ ^]^ suggesting the infiltration of T cells from the peripheral blood into the TME during ICB. The aforementioned TCR overlap between the TME and peripheral blood^[^ ^35^, ^78^, ^95^ ^]^ also implies that these intra‐tumoral treatment‐expanded T cells are blood‐related. From a dynamic perspective, novel TCR clonotypes emerge and the TCR repertoire expands during PD‐1/PD‐L1 blockade,^[^ ^120^ ^]^ indicating the mobilization of T cells from outside the TME. Tracing the dynamic of every single T cell clonotype, Yost KE, et al. discovered that the majority of post‐treatment exhausted (84%) and expanded (68%) T cells have novel TCR clonotypes absent from pre‐treatment samples.^[^ ^35^ ^]^ Similarly, post‐treatment T cell clones are predominantly composed of novel T cell clonotypes in two cohorts of BCC patients.^[^ ^39^ ^]^ In some patients, the ratio of novel clonotypes to re‐emerged clonotypes reaches ≈90% and novel T_EX_ clones account for ≈99% of total T_EX_ clones.^[^ ^39^ ^]^ The emergence of novel TCR clonotypes during PD‐1/PD‐L1 blockade is termed “clonal replacement” (Figure 3B, left panel). Conversely, it is reported in several cancer types that the majority of treatment‐expanded/post‐treatment exhausted T cells are derived from clonotypes present in pre‐treatment samples.^[^ ^39^, ^95^, ^136^ ^]^ In a study by Magen A, et al.,^[^ ^68^ ^]^ the proportion of TCRs shared between pre‐ and post‐treatment tumors was higher than that between post‐treatment tumors and either peripheral blood or LNs, suggesting a predominant local response.^[^ ^85^ ^]^ The expansion of pre‐existing TCR clonotypes induced by PD‐1/PD‐L1 blockade is termed “clonal revival”^[^ ^39^ ^]^ (Figure 3B, left panel).

Heterogeneity of immunological background may underlie the variable contributions of clonal replacement and clonal revival in different studies. The frequency of re‐emerged (pre‐existing) T_EX_ clones positively correlates with the number of baseline T_EX_ clonotypes in NSCLC, melanoma, BCC, and SCC.^[^ ^39^ ^]^ A meta‐analysis of public scRNA‐seq datasets from two NSCLC, three breast cancer, a BCC, and a SCC cohorts also demonstrated that a low level of baseline infiltration correlates with a high abundance of post‐treatment new clones.^[^ ^87^ ^]^ PD‐1/PD‐L1 blockade primarily enhances the expansion and infiltration of T cells derived from pre‐existing clonotypes in “hot” tumors, which already contain a large pool of tumor‐reactive T cells before treatment, whereas it boosts the process of novel T cell clonotypes in “cold” tumors lacking pre‐existing responding cells.^[^ ^137^ ^]^ Additionally, it has been proposed that longer intervals between treatment initiation and biopsy correlate with the emergence of novel clonotypes.^[^ ^136^ ^]^ It could be explained by that pre‐existing clonotypes expand immediately after anti‐PD‐1 treatment, while novel clonotypes take longer to arise and infiltrate the tumor,^[^ ^138^ ^]^ which aligns with the increase in peripheral tumor‐reactive CD8^+^ T cells two to four weeks after treatment initiation.^[^ ^78^, ^87^, ^95^, ^135^ ^]^ However, a follow‐up analysis of NSCLC patients receiving biopsy at different post‐treatment time points showed that it is the baseline clonotype abundance rather than the sampling time gap that underlies the frequency of novel clonotypes.^[^ ^87^ ^]^ Generally, current evidence suggests that baseline infiltration may be a dominant determinant of the clonal dynamics of T cells upon PD‐1/PD‐L1 blockade, with a lower pre‐treatment T cell abundance linking to a higher proportion of novel T cell clonotypes. Given limited samples available for analysis, future studies are expected to confirm the hypothesis and explore confounding factors such as the sampling time.

Overall, both systemic and local immune responses could be provoked by PD‐1/PD‐L1 blockade, albeit with different relative contributions. It is important to note that studies based on TCR analysis alone do not provide definitive evidence for the exact source of intra‐tumoral treatment‐expanded T cells, as T cells with pre‐existing TCR clonotypes can either expand locally or be replenished from peripheral blood. Further investigation applying robust cell tracing analysis is expected to chart T cell dynamics during treatment and evaluate the relative contributions of local expansion and peripheral replenishment. Another question that arises is the exact origin(s) of the replenished T cells from the peripheral blood. Based on the studies mentioned above, it is reasonable to conclude that these responding cells primarily, if not exclusively, originate from LNs. However, contributions from other possible sources of T cells, such as the spleen, thymus, bone marrow, and nearby healthy tissue, cannot be ruled out.

## 4 Additional PD‐1/PD‐L1 Blockade Responders

### 4.1 PD‐1/PD‐L1 Blockade Responders in CD4^+^ T Cells

As CD8^+^ T cells are widely recognized as the main fighters against cancer, most research discussed in previous sections has focused on CD8^+^ T cells or T cells in general. However, the importance of CD4^+^ T cells in anti‐tumor immunity and their role in the response to PD‐1/PD‐L1 blockade has been increasingly recognized.^[^ ^139^ ^]^

Among classical T_H_ cells, type 1 helper T (T_H1_) cells have received significant research interest in cancer.^[^ ^112^, ^140^ ^]^ T_H1_ cells, characterized by the TF T‐bet, can secrete IFNγ and TNFα, thereby activating other immune cells, such as natural killer (NK) cells and cytotoxic CD8^+^ T cells.^[^ ^141^ ^]^ Follicular helper T (T_FH_) cells play a role in both humoral immunity via stimulating the proliferation and differentiation of B cells and cellular immunity via enhancing the function of CD8^+^ T cells through IL‐21 secretion.^[^ ^141^ ^]^ T_FH_ cells are considered memory‐like. They can effectively self‐renew to maintain homeostasis without antigen stimulation^[^ ^142^, ^143^ ^]^ and are preferentially sustained under chronic antigen exposure.^[^ ^61^, ^143^, ^144^, ^145^ ^]^ In recent years, cytotoxic CD4^+^ T cells capable of secreting granzymes and perforin have been discovered in mice and humans. As they express key features of different T_H_ cell subsets, such as T_H1_ cells^[^ ^146^ ^]^ and T_FH_ cells,^[^ ^147^, ^148^, ^149^, ^150^ ^]^ further exploration is required to determine the developmental pathway and reliable markers of this specific cell subset and its relationship with conventional T_H_ classification.

Resembling CD8^+^ T cells, CD4^+^ helper T cells and cytotoxic T cells become exhausted during chronic antigen exposure, as manifested by impaired proliferation capacity, inhibited cell cycle, co‐expression of multiple IRs, and loss of helper and cytotoxic functions.^[^ ^77^, ^151^, ^152^, ^153^, ^154^ ^]^ PD‐1/PD‐L1 blockade induces expansion of T_H1_ cells,^[^ ^155^, ^156^, ^157^ ^]^ T_FH_ cells,^[^ ^35^, ^41^, ^120^, ^136^, ^158^, ^159^, ^160^ ^]^ CXCL13^+^ T_H_ cells,^[^ ^85^ ^]^ and cytotoxic CD4^+^ T cells.^[^ ^155^, ^161^ ^]^ It also strengthens T‐bet expression and restores T_H1_ prototypical cytokine production of IFNγ and TNFα.^[^ ^154^, ^155^, ^156^, ^162^ ^]^ Restored T_FH_ functions have also been reported by PD‐1/PD‐L1 blockade.^[^ ^163^, ^164^ ^]^ Additionally, gene signatures of CXCL13^+^ CD4^+^ T_H1_‐like cells^[^ ^87^, ^165^, ^166^ ^]^ and cytotoxic CD4^+^ T cells^[^ ^161^, ^167^ ^]^ are associated with the response toward PD‐1/PD‐L1 blockade.

Recent research has revealed the heterogeneity of CD4^+^ T cell subsets in chronic LCMV infection, which is in stark similarity to that of CD8^+^ T_EX_ cells.^[^ ^51^, ^168^, ^169^ ^]^ CD4^+^ progenitor/memory‐like T cells can self‐renew, differentiate into both T_FH_ cells and other effector CD4^+^ T cells, and sustain the anti‐viral immune response. Furthermore, in a mouse mesothelioma model, CD4^+^ T_EX_^prog^ cells are reported to be transcriptionally similar to CD8^+^ T_EX_^prog^ cells and increase during PD‐1/PD‐L1 blockade.^[^ ^122^ ^]^ Notably, a subset of non‐cytotoxic T_FH_ cells also exhibit a stem‐like progenitor exhaustion phenotype.^[^ ^41^ ^]^ They can differentiate into T_FH_‐like cytotoxic CD4^+^ T cells, which bear a terminally differentiated exhaustion phenotype. PD‐1 blockade can activate non‐cytotoxic progenitor T_FH_ cells but not T_FH_‐like cytotoxic cells. While there is an accumulation of correlative evidence, unlike in the case of CD8^+^ T cells, robust evidence confirming which CD4^+^ T cell subset specifically responds to PD‐1/PD‐L1 blockade is still lacking.

Another major population of intra‐tumoral CD4^+^ T cells is regulatory T (T_REG_) cells. They play a crucial role in preventing excess immune responses while also limiting anti‐tumor immunity.^[^ ^170^, ^171^ ^]^ Similar to other T cell subsets, PD‐1 expression can be induced on T_REG_ cells upon activation,^[^ ^172^ ^]^ raising the question of whether and how PD‐1/PD‐L1 blockade affects T_REG_ cells. Current studies have yielded mixed results. PD‐1 blockade has been found to impair the functional maturation of T_REG_ cells, leading to enhanced tumor control,^[^ ^173^ ^]^ while it has also been reported to enhance the proliferation and suppressive functions of T_REG_ cells, which result in hyper‐progressive diseases.^[^ ^174^, ^175^, ^176^ ^]^ The proportion of T_REG_ cells can either increase,^[^ ^156^ ^]^ decrease,^[^ ^39^, ^177^ ^]^ or remain stable^[^ ^178^ ^]^ during treatment. Notably, it is recently proposed that the increase in T_REG_ cells under PD‐1/PD‐L1 blockade may be a secondary effect of increased secretion of IL‐2 by CD8+ T cells upon treatment.^[^ ^179^ ^]^ A more in‐depth study of the response pattern of T_REG_ cells is expected.

In general, current studies have provided abundant yet preliminary phenotypic data suggesting an exhaustion‐like state acquired by CD4^+^ T cells and their response to PD‐1/PD‐L1 blockade. Further investigation can be approached from two main perspectives. First, the research outlines and methodologies used in studies of CD8^+^ T cells can be applied to CD4^+^ T cells to investigate their behavior and the underlying molecular mechanisms. Second, special attention should be given to the unique features of CD4^+^ T cells, such as their unique cell clustering and biology.

### 4.2 Other Immunocytes as PD‐1/PD‐L1 Blockade Responders

Although non‐T immunocytes are commonly known as sources of PD‐L1, they can express PD‐1 and may respond to PD‐1/PD‐L1 blockade. For example, targeted deletion of PD‐1 in myeloid progenitor cells induces more differentiated macrophages and DCs and promotes their differentiation into effector cells capable of phagocytosis and antigen presentation.^[^ ^180^ ^]^ This effect is at least partially mediated by activating ERK1/2 and mTOR pathways and rewiring cholesterol metabolism.^[^ ^180^ ^]^ Over 50% of tumor‐associated macrophages express PD‐1,^[^ ^181^ ^]^ which inhibits their phagocytic activity against cancer cells and induces an immunosuppressive M2‐like program.^[^ ^181^, ^182^, ^183^ ^]^ Correspondingly, PD‐1/PD‐L1 blockade promotes M1 polarization, improving tumor control and survival.^[^ ^181^, ^182^, ^183^ ^]^ PD‐1 blockade increases IL‐10 secretion by tumor‐infiltrating dendritic cells (TIDCs),^[^ ^184^, ^185^ ^]^ which blunts their antigen‐presenting function.^[^ ^186^ ^]^ The PD‐1 expression level on TIDCs is further augmented by the induced IL‐10 secretion, forming an immunosuppressive feedforward loop and resulting in PD‐1 blockade resistance.^[^ ^184^, ^185^ ^]^ PD‐1 expression on B cells is induced upon B‐cell receptor triggering, and it could identify a subset of regulatory B cells capable of producing IL‐10.^[^ ^187^, ^188^ ^]^ Disrupting this axis on B cells enhances their activation, proliferation, and production of inflammatory cytokines.^[^ ^187^ ^]^ Although NK cells do not intrinsically express PD‐1, they can trogocytose PD‐1 from leukemia cells via SLAM receptors when activated.^[^ ^189^ ^]^ This acquisition of PD‐1 can suppress their maturation, proliferation, degranulation, and cytokine production and promote their apoptosis, leading to impaired tumor control in mouse models and poorer patient outcomes.^[^ ^190^, ^191^ ^]^ Blockade of this axis induces a strong NK cell‐mediated immune response, which is critical to successful immunotherapy.^[^ ^190^, ^191^ ^]^ Expansion of NK cells following immunotherapy has been observed in the TME, peripheral blood, and LNs in NSCLC patients.^[^ ^157^ ^]^

### 4.3 Tumor cells as PD‐1/PD‐L1 blockade responders

PD‐1 is expressed across a broad range of tumor cells.^[^ ^192^ ^]^ However, the effects of PD‐1 on tumor cells are not yet fully understood. It has been observed in different tumor cell types that the intrinsic activity of PD‐1 inhibits the proliferation and survival of tumor cells through the activation of AKT and ERK1/2 pathways.^[^ ^193^ ^]^ Blocking this axis promotes tumor growth, which may mediate treatment resistance.^[^ ^193^ ^]^ However, contrasting results have also been reported that the PD‐1 receptor expressed on tumor cells promotes their growth via activating mTOR signaling.^[^ ^194^, ^195^ ^]^ Targeted antibodies reduce tumor growth in an immune‐independent way.^[^ ^194^, ^195^ ^]^ These findings create a “tumor cell‐intrinsic PD‐1/PD‐L1 paradox”, which may be explained by distinct signaling pathways utilized by different tumors.

Overall, there is increasing evidence supporting the existence of PD‐1/PD‐L1 blockade responders beyond T cells. However, most studies to date have only reported phenotypic, functional, and/or quantitative changes in these cells during PD‐1/PD‐L1 blockade. The correlative and implicative nature of current evidence calls for a more in‐depth characterization of the response of these non‐T cell responders. Notably, it is crucial to determine whether the observed differences are a direct effect of PD‐1/PD‐L1 blockade on these cells or a secondary effect resulting from the impact of PD‐1/PD‐L1 blockade on other components. Additionally, it is important to determine the relative contribution of responses produced by different cell types to the efficacy of anti‐PD‐1/PD‐L1 treatment.

## 5 New Perspectives on the Crosstalk between PD‐1/PD‐L1 blockade and Other Immunomodulatory Therapies

Over the past decade, numerous immunomodulatory therapeutics have emerged and are often used in combination with PD‐1/PD‐L1 blockade. The updated understanding of PD‐1/PD‐L1 blockade responders provides new insights into the interplay between components of combinatorial immunotherapy.

Cytotoxic T lymphocyte antigen‐4 (CTLA‐4) inhibitor ipilimumab is the first clinically approved immune checkpoint inhibitor (ICI) for cancer.^[^ ^196^ ^]^ CTLA‐4 is induced on activated T cells and inhibits T cell proliferation and function by competing with CD28 for the co‐stimulatory signal transmitted by B7 family proteins.^[^ ^197^, ^198^ ^]^ It is also constitutively expressed in T_REG_ cells, which sustains their suppressive function and dampens T cell priming via trans‐endocytosis‐mediated B7‐1/B7‐2 depletion and induced IDO expression in APCs.^[^ ^197^, ^198^ ^]^ The combination of ipilimumab and nivolumab induced higher objective response rates than single‐agent ICIs and has been approved for a variety of metastatic cancers.^[^ ^199^, ^200^ ^]^ However, there is no clear consensus on the underlying mechanisms of the potential synergy. Current study of the impact of CTLA‐4 blockade on PD‐1/PD‐L1 blockade responders is limited. As the CTLA‐4/B7 axis mainly functions in LNs and is implicated in T cell priming, an important process impacting T cell exhaustion and response to PD‐1/PD‐L1 blockade,^[^ ^201^, ^202^, ^203^ ^]^ it is reasonable to hypothesize that CTLA‐4 blockade synergizes with PD‐1/PD‐L1 blockade via promoting LN‐resident T_TSM_ cells and T_EX_^prog^ cells. Pertinently, CTLA‐4 has been reported to inhibit the effector and proliferative abilities of memory CD8^+^ T cells.^[^ ^204^ ^]^ Blocking CTLA‐4 during CD8^+^ T cell priming leads to increased expansion and maintenance of antigen‐specific memory CD8^+^ T cells, resulting in enhanced protective immunity against bacterial infections.^[^ ^205^ ^]^ Expansion of T_EX_^prog^ cells upon combined anti‐CTLA‐4 and anti‐PD‐1 treatment has been observed in a mouse liver cancer model, although the effects of combined ICIs and single‐agent ICIs were not compared.^[^ ^206^ ^]^ It is also important to determine whether the expansion comes from 1) enhanced proliferation of upstream progenitors, such as T_TSM_ cells, or T_EX_^prog^ cells themselves, or 2) decreased terminal exhaustion. In addition, as the crosstalk between CD8^+^ T cells and T_REG_ cells can negatively regulate response to PD‐1/PD‐L1 blockade,^[^ ^179^ ^]^ anti‐CTLA‐4 antibodies may indirectly enhance the efficacy of PD‐1/PD‐L1 blockade via depleting T_REG_ cells.^[^ ^197^, ^198^ ^]^ Nonetheless, future preclinical and clinical studies are expected to elucidate the immunological landscape under combined PD‐1/PD‐L1 and CTLA‐4 inhibition.

Beyond the classical PD‐1/PD‐L1 and CTLA‐4/B7 pathways, other IRs on T cells have been targeted and tested in clinical settings. Co‐expression of multiple checkpoints is associated with exhaustion severity,^[^ ^207^, ^208^ ^]^ suggesting that blocking multiple IRs concurrently or subsequently might prevent exhaustion, maintain stemness, and enhance the therapeutic response to PD‐1/PD‐L1 blockade. Moreover, the distinct expression patterns of IRs on different T cell subsets offer the possibility of activating non‐responsive cells to PD‐1/PD‐L1 blockade. For example, Tim3 is exclusively expressed on T_EX_^term^ cells but not on T_EX_^prog^ cells.^[^ ^34^, ^51^, ^60^, ^63^, ^64^, ^80^, ^209^, ^210^ ^]^ LAG3 is a reliable marker for terminally differentiated CD4^+^ T_FH_‐like cytotoxic cells (CD4^+^ T_EX_ cells).^[^ ^41^ ^]^ Correspondingly, standalone PD‐1 blockade only activates CD4^+^ T_FH_‐like progenitor cells, while dual LAG3 and PD‐1 blockade activates both groups.^[^ ^41^ ^]^ Many studies have demonstrated the therapeutic synergy between additional IR blockade and PD‐1/PD‐L1 blockade.^[^ ^211^, ^212^, ^213^, ^214^, ^215^, ^216^ ^]^ However, direct evidence linking this synergy to specific responder subsets is currently lacking.

Co‐stimulation is crucial for regulating T‐cell activation, differentiation, proliferation, and function.^[^ ^217^ ^]^ Similar to additional IRs, targeting co‐stimulatory molecules enhances the efficacy of PD‐1/PD‐L1 blockade.^[^ ^218^, ^219^ ^]^ This synergy is likely achieved by either expanding a larger pool of stem‐like T cells or reinvigorating terminally differentiated T cells that do not respond to PD‐1/PD‐L1 blockade. 4‐1BB (CD137, TNFRSF9) agonism promotes both proliferation and terminal differentiation of T_EX_ cells, leading to intra‐tumoral T cell expansion and enhanced effector function.^[^ ^220^, ^221^, ^222^, ^223^ ^]^ It is specifically upregulated in terminally differentiated PD‐1^high^ CD39^+^ TILs and barely detected on T_EX_^prog^ cells.^[^ ^222^, ^224^ ^]^ Correspondingly, 4‐1BB agonism mainly affects terminally differentiated TILs independent of progenitor ones.^[^ ^224^ ^]^ OX40 (CD134, TNFRSF4) agonists also synergize with PD‐1/PD‐L1 blockade in promoting T cell proliferation and effector function.^[^ ^225^, ^226^ ^]^ In contrast to 4‐1BB, OX40 agonism induces a higher level of CD62L^+^ T_MEM_ cells and alleviates T‐cell exhaustion.^[^ ^226^ ^]^ The triplet combination of anti‐PD‐1 treatment and OX40/4‐1BB agonism expands a stem‐like PD‐1^lo^ KLRG‐1^+^ Ki‐67^+^ CD8^+^ T cell subpopulation rather than reinvigorating terminally exhausted CD8^+^ T cells, enhancing the efficacy of PD‐1/PD‐L1 blockade.^[^ ^227^ ^]^ Besides, other co‐stimulatory pathways are also worth investigating.^[^ ^217^ ^]^

Cytokines play a critical role in regulating T cell phenotype.^[^ ^228^ ^]^ High levels of IL‐2 drive terminal differentiation and increase effector functions, while low levels of IL‐2 promote the development of stem‐like T cells.^[^ ^229^, ^230^, ^231^, ^232^ ^]^ In addition to the accumulation of stem‐like T cells,^[^ ^233^, ^234^ ^]^ incorporating IL‐2 into PD‐1/PD‐L1 blockade also induces a skewed differentiation from stem‐like CD8^+^ T cells toward a distinct subset of effector CD8^+^ T cells.^[^ ^86^, ^235^ ^]^ Similarly, IL‐10 maintains the TCF1^+^ CD8^+^ T cell population^[^ ^236^ ^]^ while promoting the expansion and effector function of T_EX_^term^ cells independent of T_EX_^prog^ cells.^[^ ^237^ ^]^ IL‐33, IL‐21, and IL‐15 also preserve the stemness of TCF1^+^ T cells in chronic viral infection.^[^ ^238^, ^239^, ^240^ ^]^ Overall, manipulating T cell response through cytokines during PD‐1/PD‐L1 blockade is a promising approach to overcome treatment resistance,^[^ ^241^, ^242^ ^]^ especially considering the large family of cytokines and their diverse functions. Nevertheless, joint preclinical and clinical efforts are required to determine the optimal combinations.

Other regulatory factors of PD‐1/PD‐L1 blockade responders have been discovered, highlighted by epigenetic and metabolic factors.^[^ ^243^ ^]^ The TF TOX coordinates the epigenetic imprinting of T cell exhaustion, promoting the exhaustion phenotype and supporting the survival of these dysfunctional cells.^[^ ^244^, ^245^, ^246^, ^247^ ^]^ TCF1 is a well‐established marker for T_EX_^prog^ cells, and it is both necessary and sufficient to support the T_EX_^prog^ population via induction of Bcl6.^[^ ^51^ ^]^ A recent study discovered other three key TFs regulating the transition between subsets by CRISPR screens: 1) IKAROS inhibiting the transition from resting T_EX_^prog^ cells to actively proliferating T_EX_^prog^ cells, 2) ETS1 promoting the transition from active T_EX_^prog^ cells to early T_EX_^term^ cells, and 3) RBPJ suppressing terminal exhaustion.^[^ ^84^ ^]^ Other important TFs include STAT3,^[^ ^83^ ^]^ IRF4 and BATF,^[^ ^248^, ^249^, ^250^ ^]^ T‐bet and Eomes,^[^ ^37^ ^]^ MYB,^[^ ^54^ ^]^ and BACH2,^[^ ^251^ ^]^ each displaying unique dynamics along the exhaustion process. Metabolites, metabolic intermediates, and metabolic enzymes also influence T‐cell differentiation. Decreased mitochondrial fitness drives terminal differentiation.^[^ ^243^, ^252^, ^253^ ^]^ T_EX_^prog^ cells preferentially rely on mitochondrial fatty acid oxidation and oxidative phosphorylation, while T_EX_^term^ cells mainly rely on glycolysis and oxidative phosphorylation.^[^ ^254^, ^255^, ^256^ ^]^ Lactate accumulated in the TME preserves the stemness of CD8^+^ T cells,^[^ ^257^ ^]^ while an increased lipid uptake is correlated with terminal exhaustion.^[^ ^59^, ^258^ ^]^ Despite the significant therapeutic potential suggested by mechanistic studies, therapeutic attempts in this area are still rare.

Antigen properties shape antigen‐specific T cell phenotype.^[^ ^259^, ^260^, ^261^ ^]^ Thus, antigen‐based cancer vaccines may be utilized to induce stem‐like T cells to enhance PD‐1/PD‐L1 blockade response. Vaccination could eliminate a dysfunctional TCF1^+^ progenitor T cell subset targeting subdominant antigens and improve response to ICB.^[^ ^260^ ^]^ Adding a gorilla adenovirus vaccine targeting tumor neoepitopes to anti‐PD‐1 treatment accumulates TCF1^+^ stem‐like T cells in TdLNs and T_EFF_ cells in tumors, and expands neoantigen‐specific CD8^+^ T cell repertoire.^[^ ^262^ ^]^ Moreover, recent findings suggest that only poorly immunogenic tumors rely on TCF1 for optimal activation of stem‐like CD8^+^ T cells in TdLNs and for the therapeutic response to PD‐1/PD‐L1 blockade, and vaccination rescues defective ICB response via enhancing tumor immunogenicity.^[^ ^229^ ^]^

Gene therapy to modulate gene expression permanently by genome editing or temporally by RNA is also immunomodulatory. As mentioned previously, antigens, cytokines, and key genes can influence the phenotype of T cells and potentially the response to PD‐1/PD‐L1 blockade. mRNA vaccines can encode tumor antigens, immunostimulatory cytokines, and Cas9 protein for genome editing.^[^ ^263^ ^]^ Cell‐intrinsic inhibitory signals transmitted by IRs or immunosuppressive cytokines can be blocked by RNA interference.^[^ ^264^, ^265^ ^]^ Therefore, although not directly associated with T_EX_^prog^ cells and T_TSM_ cells, gene therapy may represent an ideal platform for augmenting PD‐1/PD‐L1 blockade efficacy.

In addition to the therapies that synergize with PD‐1/PD‐L1 blockade via regulating T cell phenotype, the notion that responding T cells are activated in TdLNs supports the use of therapies enhancing T cell trafficking and infiltration. For example, simultaneous blockade of tumor growth factor β (TGFβ) and vascular endothelial growth factor (VEGF), which respectively impede T cell infiltration by promoting peritumoral collagen production and tumor angiogenesis, significantly enhances the immune cell infiltration and sensitizes tumors to anti‐PD‐1 treatment.^[^ ^266^ ^]^

## 6 Implications for Translational and Clinical Research

### 6.1 Improving PD‐1/PD‐L1 Blockade‐centered Therapies

**Figure 4:**
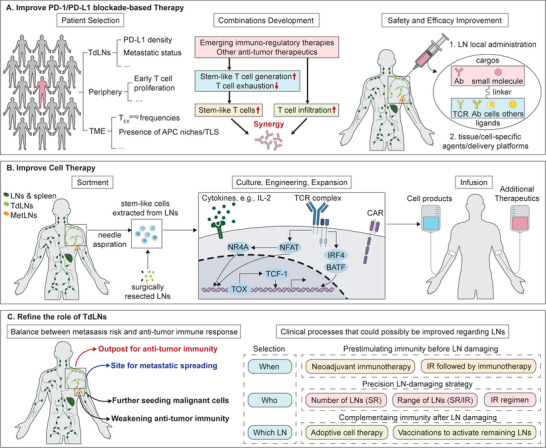
Translational and Clinical Implications of the Cellular Response to Programmed Death 1 (PD‐1)/Programmed Death Ligand 1 (PD‐L1) Blockade. A) The safety and effectiveness of PD‐1/PD‐L1 blockade‐based therapy could be improved by identifying better predictors for patient response, exploring the most promising treatment combinations, and developing targeted drug delivery strategies. B) Cell therapy can be improved by isolating stem‐like tumor‐specific T cells in tumor‐draining lymph nodes (TdLNs) and/or endowing sorted cells with more stem‐like properties during in vitro processing. The utilization of combination therapies can help preserve the stemness of the infused cell products. C) The dual role of lymph nodes (LNs) necessitates a balance between eliminating metastasized tumor cells and preserving anti‐tumor immunity when using LN‐destroying treatment. Further research is required to determine the optimal number and types of LNs to resect, the appropriate range and dosage of LN radiation, and the ideal sequencing of LN‐destroying treatment with other immunotherapies. Metastatic lymph node (MetLN), T cell receptor (TCR), antibody (Ab), chimeric antigen receptor (CAR), surgical resection (SR), irradiation (IR), progenitor exhausted T (T_EX_^prog^), antigen‐presenting cell (APC), tertiary lymphoid structures (TLS), tumor microenvironment (TME).

The identification of stem‐like T_EX_^prog^ cells and T_TSM_ cells in LNs as major responders to PD‐1/PD‐L1 blockade explains the suboptimal performance of current TME‐based predictors^[^ ^10^ ^]^ and offers insights into identifying better predictors (**Figure** **4**A, left panel). Factors that restrain the ability of LNs to generate an effective anti‐tumor immune response, such as a high density of PD‐1/PD‐L1 interaction^[^ ^119^, ^122^ ^]^ and the presence of metastasized malignant cells,^[^ ^66^ ^]^ correlate with patients’ response to ICB. In addition, T cell proliferative response early after treatment in the peripheral blood can help distinguish responsive patients.^[^ ^102^, ^130^, ^132^ ^]^ Plausible parameters include the number/proportion of antigen‐specific T cells, the expression level of proliferation markers, and changes in the TCR repertoire diversity. In a neoadjuvant setting, the frequency of activated blood CD8^+^ T cells in the blood, particularly the less‐differentiated PD‐1^+^ KLRG1^‐^ CD8^+^ T cells, is strongly associated with the patients’ pathological response.^[^ ^95^ ^]^ Given that T cell local expansion also accounts for a substantial proportion of cellular response to PD‐1/PD‐L1 blockade, it is also worth considering intra‐tumoral progenitor‐related features for response prediction. For example, in esophageal cancer patients receiving neoadjuvant PD‐1 blockade, the T_EX_^prog^ cell signature score has shown higher predictive sensitivity and specificity for treatment response compared to PD‐L1 expression.^[^ ^267^ ^]^

As discussed in the previous section, many emerging immunomodulatory therapies are likely to synergize with PD‐1/PD‐L1 blockade via expanding responders or rewiring non‐responders (Figure 4A, middle panel). Promising examples include therapies targeting additional IRs, co‐stimulatory molecules, and cytokines, cancer vaccines, gene therapy, and therapies promoting T cell homing and infiltration. The knowledge gained from understanding the identity of PD‐1/PD‐L1 blockade responders and their response dynamics can serve as a clue for identifying optimal combinations.

Targeted delivery to responders in a tissue‐specific and/or cell‐specific manner is promising to improve the performance of anti‐PD‐1/PD‐L1 agents (Figure 4A, right panel). Preclinical studies have shown that intradermal administration and low‐dose intrapleural administration (1/80 of systemic dose) enable the preferential accumulation of ICIs in TdLNs compared to systemic administration, and have demonstrated comparable anti‐tumor effects in three different subcutaneous tumor models.^[^ ^122^, ^123^ ^]^ Similar TdLN‐targeting effect and TME reprogramming can also be achieved by nanoneedle‐based transdermal administration of anti‐PD‐1 antibodies in a mouse melanoma model.^[^ ^268^ ^]^ Currently, both intravenous and subcutaneous administration have been approved for different ICIs, with equivocal safety and efficacy. The feasibility of these local delivery strategies applied in animal studies in clinical settings remains to be investigated. Nanotechnology represents a useful platform enabling the selective accumulation of ICIs in the TME. For instance, pH‐responsive^[^ ^269^ ^]^ and ROS‐responsive^[^ ^270^ ^]^ nanocarriers of anti‐PD‐L1 therapeutics induced selectively higher concentrations in the TME and better therapeutic efficacy. Chimeric antigen receptor (CAR) T cells can also be engineered to secrete PD‐1/PD‐L1 blocking molecules at tumor sites.^[^ ^271^, ^272^, ^273^, ^274^ ^]^ T cell‐based delivery has demonstrated superior anti‐tumor efficacy and safety than combined cell transfer and checkpoint blockade.^[^ ^271^, ^272^, ^273^, ^274^ ^]^ Whether these TME‐targeted delivery techniques can be extrapolated into the targeted delivery of ICIs to LNs requires further exploration. On the other hand, to avoid the potentially immunosuppressive response mediated by additional responders, such as T_REG_^[^ ^174^ ^]^ and tumor cells,^[^ ^193^ ^]^ cell‐specific agents targeting the PD‐1 receptor on a specific cell subset have the potential to enhance treatment efficacy. In the context of autoimmune diseases, researchers have engineered a bispecific molecule consisting of a PD‐1 agonist and a recombinant TCR.^[^ ^275^ ^]^ It remains inactive in its soluble form, capable of activating the PD‐1 receptor on activated T cells localized to pancreatic β cells and inducing localized immune suppression without impairing systemic immunity in type I diabetes patients.^[^ ^275^ ^]^ Antibody‐modified nanoparticles are ideal tools for cell type‐specific delivery. In vivo targeted reprogramming of CAR T cells could be achieved through CD3‐targeting nanoparticles.^[^ ^276^ ^]^ CD8 T cell‐targeted nanoparticles encapsulating immunostimulatory agents demonstrate higher efficacy in tumor growth control and prolonging survival.^[^ ^277^ ^]^ Notably, targeting functional markers, such as PD‐1, can also be achieved.^[^ ^277^ ^]^ These discoveries have the potential to be applied in targeting specific responders of immunotherapies, calling for future research endeavors.

### 6.2 Improving Cell Therapy

Utilizing cells with stem‐ and memory‐like properties holds the potential to enhance the persistence and efficacy of cell therapy. The proportion of CD39^‐^ CD69^‐^ stem‐like TILs in the TIL products correlates with complete cancer regression and TIL persistence in melanoma patients. Transfer of CD39^‐^ CD69^‐^ cells into tumor‐bearing mice yielded significantly better tumor control than transferring CD39^+^ CD69^+^ cells, even at a tenfold concentration.^[^ ^82^ ^]^ Similarly, characteristics of less‐differentiated CAR T cell products positively correlate with long‐term anti‐tumor activity and clinical response.^[^ ^278^ ^]^ Stem‐like CAR T cells have shown better leukemia control while reducing adverse events.^[^ ^279^ ^]^

Strategies such as the in vitro induction or selection of stem‐like cell products^[^ ^280^ ^]^ can be employed to improve current cell therapy (Figure 4B). Given that LNs harbor T_TSM_ cells and T_EX_^prog^ cells, cell products derived from cells sorted from LNs may outperform current cell products derived from either tumor (e.g., TIL therapy) or peripheral blood (e.g., CAR immune cell therapy, TCR‐T cell therapy). In addition, concurrent use of PD‐1/PD‐L1 blocking agents and reprogramming exhaustion‐related epigenetic and metabolic shifts may antagonize T cell exhaustion and maintain the progenitor phenotype in vivo.^[^ ^281^ ^]^

### 6.3 Improving Management of TdLNs

By analyzing the influence of TdLN dissection,^[^ ^40^, ^66^ ^]^ blocking cell migration from LNs pharmacologically,^[^ ^119^, ^122^ ^]^ and TdLN‐targeted ICB^[^ ^40^, ^122^, ^123^ ^]^ on the therapeutic efficacy of ICIs, multiple research teams have demonstrated the importance of TdLNs as the basis of anti‐tumor immune response. Consistently, LN‐damaging strategies that aim at lowering the risk for metastases, such as surgical removal^[^ ^282^ ^]^ or irradiation,^[^ ^283^, ^284^ ^]^ impair anti‐tumor immunity (Figure 4C, left panel). The radiotherapy‐induced abscopal effect, which refers to the anti‐tumor effect observed in distant, non‐irradiated tumors due to systemic immune awakening, is also reduced by LN irradiation.^[^ ^126^, ^285^ ^]^ Therefore, the management of TdLNs requires a balance between lowering the risk of metastasis and preserving their potential to generate anti‐tumor immune response (Figure 4C, left panel).

Immunotherapy before surgical removal of TdLNs is capable of pre‐stimulating anti‐tumor immune response and preserves enough numbers of activated T cells to function against remaining tumors after LN removal (Figure 4C, right panel). Preoperative immunotherapy resulted in a higher number of related immune cells in resected tumor tissue and patients’ blood, which persisted till at least one month post‐surgery.^[^ ^66^ ^]^ This approach, mostly combined with concurrent chemotherapy or chemoradiotherapy, is being investigated in various cancer types, and preliminary results are promising.^[^ ^286^ ^]^ Sequencing lymphatic‐preserving radiation with immunotherapy is another feasible approach to address this challenge. Sequential LN‐sparing radiation and ICB induce an optimal and durable treatment response that controls both the primary and metastatic diseases and prevents tumor rechallenge.^[^ ^125^, ^284^, ^287^ ^]^ Although LN‐sparing radiation may increase the regional recurrence rate (Figure 4C, right panel), receiving subsequent surgical resection or including sentinel LNs into the irradiation field may reverse this effect, enabling both the activation of T cell response and the elimination of metastatic tumor cells.^[^ ^283^ ^]^ In addition to pre‐stimulating anti‐tumor immunity before LN damage by radiation or surgery, cancer vaccination capable of activating remaining LNs or adoptive cell therapy to directly provide tumor‐specific T cells are also promising to complement immune response.

The dose of radiation plays a critical role in its immunological effects. Low‐dose radiotherapy (LDRT) can reprogram the TME, turning an immunologically “cold” tumor “hot”.^[^ ^288^ ^]^ Whether LDRT of LNs can also activate anti‐tumor immunity in LNs or rescue the ability of metastasized LNs (MetLNs) to generate immune response is worth investigating (Figure 4C, right panel). Preliminary clinical results of combined ICB and neoadjuvant LDRT delivered only to the primary tumor and metastatic LNs in head and neck squamous cell carcinoma are promising,^[^ ^289^, ^290^ ^]^ although the specific contribution of LN‐directed LDRT needs further exploration. MetLNs display a larger fraction occupied by T_EX_^term^ cells, indicating a failure of T_EX_^prog^ cells to become activated and differentiate into T_EX_^term^ cells. They also have a higher number and activity of T_REG_ cells after ICI treatment than non‐metastasized LNs.^[^ ^66^ ^]^ This suggests that the immune activation induced by PD‐1/PD‐L1 blockade is impaired in MetLNs, and selective surgical removal or irradiation strategies may be finetuned based on the status of LNs (Figure 4C, right panel).

Overall, further investigation is needed to test the viability of these theoretical conjectures in animal models and humans. It is also important to determine whether these preclinical findings can be extrapolated to the clinical setting.

## 7 Conclusion

In this review, we summarize current knowledge regarding the identity, spatial distribution, and treatment‐induced dynamics of cells responding to PD‐1/PD‐L1 blockade, with a focus on CD8^+^ T cells. Anti‐tumor CD8^+^ T cell response is mainly produced by highly heterogeneous T_EX_ cells and both circulating and tissue‐resident memory T cells. T_EX_ cells progress from a stem‐like T_EX_^prog^ state to an effector‐like T_EX_^term^ state. Among all T_EX_ subsets, T_EX_^prog^ cells are the most responsive to PD‐1/PD‐L1 blockade. Although it is conventionally recognized that chronic infection and cancer preclude memory formation, T_TSM_ cells have been recently identified in TdLNs. T_TSM_ cells are not exhausted nor inferior to conventional T_MEM_ cells regarding memory potential and are considered to be bona fide responders to PD‐1/PD‐L1 blockade superior to T_EX_^prog^ cells. T_RM_ cells also show preliminary evidence of responding to PD‐1/PD‐L1 blockade. In addition to cellular identity, the spatiotemporal dynamics of responding T cells during treatment have been gradually unveiled in recent years. Supported by the close link between LNs and PD‐1/PD‐L1 blockade‐responding cells, peripheral T cell proliferation observed in patients receiving ICB, and the emergence of novel intra‐tumoral TCR clonotypes, it is now recognized that PD‐1/PD‐L1 blockade activates both systemic and local T cell responses, leading to T cell clonal replacement and revival during treatment. Beyond the extensively studied CD8^+^ T cell compartment, CD4^+^ T cells, non‐T immune cells, and tumor cells also show numerical and/or functional changes after PD‐1/PD‐L1 blockade, implying their roles as potential responders of PD‐1/PD‐L1 blockade.

These updated insights present a wide research space and open new avenues for improving immune‐related cancer treatment. Translational efforts and integration of bench and bedside investigations are required to translate these cutting‐edge mechanistic discoveries into real‐time clinical benefits for patients. Further basic studies, with the help of elegantly designed experiments and solid in silico analysis, are also crucial to fully elucidate the T cell dynamics during tumor development and PD‐1/PD‐L1 blockade.

In conclusion, PD‐1/PD‐L1 blockade is an area with great potential, which has revolutionized anti‐tumor immunotherapy and become increasingly vital, albeit with many challenges to overcome. Immense efforts have been devoted to unveiling the cellular response to PD‐1/PD‐L1 blockade and significant progress has been made. The emerging paradigm suggesting that stem‐like T cells outside the TME are the main responders to PD‐1/PD‐L1 blockade holds hopeful promise in ameliorating current challenges and ushering tumor immunotherapy into a new chapter.

## Conflict of Interest

The authors declare no conflict of interest.
